# In Search of Better Spermatogonial Preservation by Supplementation of Cryopreserved Human Immature Testicular Tissue Xenografts with *N*-acetylcysteine and Testosterone

**DOI:** 10.3389/fsurg.2014.00047

**Published:** 2014-12-02

**Authors:** Jonathan Poels, Gaël Abou-Ghannam, Sophie Herman, Anne Van Langendonckt, François-Xavier Wese, Christine Wyns

**Affiliations:** ^1^Gynecology Unit, Medical School, Institut de Recherche Expérimentale et Clinique, Université Catholique de Louvain, Brussels, Belgium; ^2^Department of Gynecology-Andrology, Cliniques Universitaires Saint-Luc, Brussels, Belgium; ^3^Department of Urology, Cliniques Universitaires Saint-Luc, Brussels, Belgium

**Keywords:** cryopreservation, fertility preservation, immature testicular tissue, spermatogonia, supplemented xenografting

## Abstract

Controlled slow-freezing is the procedure currently applied for immature testicular tissue (ITT) cryobanking in clinical practice. Vitrification has been proposed as a promising alternative, with a view to better preserve the spermatogonial stem cells for future fertility restoration by autografting in young boys suffering from cancer. It appears that besides the potential influence of the cryopreservation technique used, the transplantation procedure itself has a significant impact on spermatogonial loss observed in ITT xenografts. Eighteen ITT pieces issued from 6 patients aged 2–15 years were used. Fragments of fresh tissue (serving as ungrafted controls), frozen-thawed tissue, frozen-thawed tissue supplemented with *N*-acetylcysteine (NAC), and frozen-thawed tissue supplemented with testosterone xenografted to nude mice for 5 days were compared. Upon graft removal, histological and immunohistochemical analyses were performed to evaluate spermatogonia, intratubular proliferation, and intrinsic and extrinsic apoptosis. A significant decrease in the integrity of intact seminiferous tubules was found in all three grafted groups. Spermatogonia were observed by immunohistochemistry in all grafted groups, with recovery rates of 67, 63, and 53%, respectively, for slow-frozen tissue, slow-frozen tissue supplemented with NAC, and slow-frozen tissue supplemented with testosterone. Apoptosis evidenced by active caspase-3 and terminal deoxynucleotidyl transferase-mediated dUTP nick end labeling was similar in all grafts. The study is limited by the low availability of ITT samples of human origin, and no clear impact of graft supplementation was found. The mouse xenotransplantation model needs to be refined to investigate human spermatogenesis in human ITT grafts.

## Introduction

Gonadotoxic treatments used for childhood cancer therapy can irreversibly affect fertility in adulthood. With increased effectiveness of these cancer treatments, ≥80% of patients survive their disease (1–3), so finding techniques to preserve fertility has become a priority quality-of-life issue. While sperm can be cryopreserved in adolescent boys and adults, this is not an option for young boys, since no spermatozoa are produced before puberty. However, cryopreservation of immature testicular tissue (ITT) containing spermatogonial stem cells (SSCs) can be offered to these prepubertal patients, with a view to future autotransplantation or *in vitro* maturation.

In human beings, evaluation of cryopreservation techniques for ITT has been done through a mouse xenotransplantation model. Although studies show promising results with survival of spermatogonia (SG) and initiation of spermatogenesis, the recovery rate of these SG appears to be greatly reduced, regardless of grafting site or follicle-stimulating hormone (FSH) supplementation (4–8). Moreover, SG loss was found to increase over time, with SG recovery rates of 14.5 and 3.7%, at 3 weeks and 6 months, respectively, for slow-frozen/thawed and xenografted tissue (4, 5).

Other methods like vitrification, which could minimize cell and tissue damage due to ice crystal formation inherent to the slow-freezing technique, could be more efficient at preserving SG.

In an attempt to improve cryopreservation techniques for SG preservation, slow-freezing and vitrification of ITT were compared after xenografting. Interestingly, both cryopreservation protocols resulted in similar SG survival rates (8), suggesting that etiologies other than the cryopreservation procedure may be implicated in SG loss. Oxidative stress due to hypoxia related to the avascular xenografting technique and/or an inadequate endocrine or paracrine host environment may be involved.

Indeed, ischemic stress can lead to tissue apoptosis or necrosis in grafts, as seen in transplanted ovarian tissue (9). Extensive apoptosis enhanced with active caspase-3 and increased numbers of terminal deoxynucleotidyl transferase-mediated dUTP nick end labeling (TUNEL)-positive cells were also previously observed in ITT transplanted for 3 days [unpublished data, Wyns, PhD thesis (10)]. It was established that during revascularization of tissue grafts, hypoxia occurs during the first 5 days, followed by progressive reoxygenation after this period (11). Addition of antioxidants to protect the graft during these first 5 days has, therefore, been considered to limit oxidative stress before revascularization.

*N*-acetylcysteine (NAC) is a good antioxidant candidate, having been used in clinical practice since 1970 as an antidote to acetaminophen intoxication (12), as well as in other clinical settings such as cancer, chronic obstructive pulmonary disease, cystic fibrosis, some infections, and contrast-induced nephropathy (13–15). *N*-acetylcysteine acts as an antioxidant preventing apoptosis by replenishing intracellular glutathione (GSH). By regenerating the pool of GSH, NAC protects cells from oxidative stress caused by reactive oxygen species (ROS) and reduces cell membrane lipid peroxidation. A protective effect of NAC on the tissue in case of testicular torsion/distortion (16–18) and apoptosis inhibition in germ cell cultures (19) have been shown.

The role of the hormonal environment for maintenance and development of the graft was also found to be key, emphasized by the finding that grafted tissue endocrine function is extrinsically modulated by the recipient mouse hypothalamic–pituitary–gonadal axis (20). However, the benefits of gonadotropin administration appear to be controversial. While administration of exogenous gonadotropins to mice led to improved tissue maturation and differentiation in pig and monkey xenografts (21, 22), this beneficial effect was not observed in xenografted testicular tissue from marmosets or horses (23, 24), nor cryopreserved and grafted human ITT (7). The influence of testosterone on xenograft outcome has not yet been investigated, even though testosterone is a key paracrine factor in spermatogenesis (25) and was found to reduce apoptosis of germ cells in cell cultures (26).

The objective of our study was, therefore, to evaluate the effect of the addition of NAC or testosterone to human slow-frozen ITT xenografts in terms of SG recovery, cell proliferation, and apoptosis.

## Materials and Methods

### Study design

Eighteen ITT pieces (1 mm^3^) were obtained from six prepubertal patients (2–15 years of age). The tissues pieces were slow-frozen, thawed, and allocated to three different mouse xenografting groups: (A) control, (B) supplemented with NAC, and (C) supplemented with testosterone and FSH. Fragments of fresh tissue fixed in a 4% paraformaldehyde (PFA 4%, VWR Chemicals, Leuven, Belgium) solution served as ungrafted controls.

In group A, the mice received 200 μl of phosphate-buffered saline (PBS, Sigma Aldrich, Bornem, Belgium) by intraperitoneal (IP) injection on day 0 and day 2. In group B, the tissue was placed in a bath (5 ml) of Hank’s balanced salt solution (HBSS, Gibco, Merelbeke, Belgium) with 30 mg of NAC (Sigma Aldrich) for 5 min at 4°C before grafting. On day 2, the mice received an IP injection of 200 μl of NAC (150 mg/kg/bodyweight). In group C, the mice were given testosterone (Fagron, Waregem, Belgium) (5 mg/kg) by intramuscular (IM) injection on day 0 and day 2 after dilution in vegetal oil, sterilized by filtration. As exogenous testosterone administration induces inhibition of the hypothalamic–pituitary–gonadal axis by negative feedback, 1.5 IU FSH (27) was concomitantly administered to maintain Sertoli cell stimulation. After 5 days, the grafts were recovered and fixed in PFA 4% solution, embedded in paraffin, and cut into serial sections for analysis by histology and immunohistochemistry.

### Animals

Nine NMRI nu/nu mice (Janvier Laboratories, Le Genest-St-Isle, France) aged 8 weeks were used as recipients for the xenografts. They were housed in cages under filtered hoods (MicroIsolator, Uno) in rooms maintained at an ambient temperature between 22 and 24°C with a day/night cycle of 12 h. All housing material and food were autoclaved before use. The mice were fed *ad libitum* on laboratory chow (complete food for rats and mice; Pavan Carfil) and acidified water. All experiments in this study were approved by the Ethics Review Board and the Committee on Animal Research of the Catholic University of Louvain.

### Donor testicular tissue

Immature testicular tissue was retrieved from six boys aged between 2 and 15 years (2, 2, 8, 9, 14, and 15 years) after obtaining informed consent from the parents and the child’s ascent (where applicable). Sample size was small because of the scarcity of human ITT. Patients were referred by pediatric oncologists or hematologists to a reproductive specialist in fertility preservation when they considered that the risk of infertility due to treatment was high and/or the parents specifically requested fertility preservation techniques. All donors were scheduled for testicular biopsy prior to gonadotoxic treatment. Unilateral testicular sampling of less than 5% of total testicular volume (based on theoretical size by age from 0 to 12) (28) was performed by a pediatric urologist through a scrotal incision. The ethics committee agreed to testicular biopsy for research purposes only when testicular surgery was required for the child’s fertility preservation and after obtaining informed consent.

Testicular tissue was transferred in HBSS on ice to the laboratory. It was manually dissected and cut into pieces. The majority of the collected tissue was used for fertility preservation purposes. For each donor, a small piece (±1 mm^3^) fixed in PFA 4% solution (sent to the laboratory of anatomopathology) served as an ungrafted control. One fragment of ITT (±1 mm × 1 mm × 3 mm) from each boy was used for our experiment and divided into three pieces (±1 mm^3^) allocated to the three grafting groups.

### Slow-freezing and thawing

The slow-freezing protocol applied was previously described by Wyns et al. (4). Briefly, tissue pieces were placed in 1 ml freezing medium with dimethyl sulfoxide 0.7 M (DMSO, Sigma Aldrich) and sucrose 0.1 M (Sigma Aldrich) at 4°C in a 2 ml cryovial (Nunc, Denmark). Using a controlled freezer (Minicool 40 PC Air Liquide, Marne-la-Vallée, France), the vials were maintained at 0°C for 9 min, cooled at a rate of −0.5°C/min to −8°C and then held for 5 min before seeding manually at −8°C. After holding for a further 15 min at −8°C, a cooling rate of −0.5°C/min was used from −8°C to −40°C before final dehydration for 10 min at −40°C. After cooling at −7°C/min to −80°C, the vials were transferred to liquid nitrogen (−196°C). For thawing, the cryopreserved tissue was kept for 2 min at room temperature, thawed in a water bath at 37°C for 2 min, and then washed three times in a reversed sucrose concentration gradient solution (0.1, 0.05, and 0 M sucrose) for 5 min per bath, using HBSS medium on ice.

### Xenografting

The mice were anesthetized by IP injection of ketamine (75 mg/kg; Anesketin, Eurovet, Heusden-Zolder, Belgium) and medetomidine (1 mg/kg; Domitor, Pfizer, CA, USA) dissolved in PBS. They underwent bilateral castration and, in the course of the same surgery, a ±1 mm^3^ piece of donor testicular tissue was grafted without vascular anastomosis into the scrotum, according to a previously described procedure (4). After surgery, anesthesia was reversed by injection of atipamezole (1 mg/kg; Antisedan, Pfizer). Analgesia was provided by buprenorphine (0.1 mg/kg, Temgesic, Schering Plough, Kenilworth, NJ, USA) on the day of surgery and the following day. In group B, the fragment was placed in a supplementary bath containing HBSS + 30 mg NAC for 5 min before transplantation. In group C, the mice were given an injection of testosterone (5 mg/kg) and 1.5 IU FSH before reversing anesthesia.

### Graft recovery

After 5 days, the mice were anesthetized by IP injection of ketamine and euthanized by intracardiac blood puncture. The grafts were recovered and directly fixed in PFA 4% solution. The totality of the grafted tissue was used for analysis.

### Histological and immunohistochemical analysis

#### Histology

After fixation in PFA 4% solution, tissue samples were embedded in paraffin and cut into 5 μm-thick serial sections. One section every 50 μm was stained with hematoxylin-eosin (HE) for histological evaluation by light microscopy. Subsequent sections were mounted on Superfrost Plus slides and used for immunohistochemistry. Digital images were captured with a Mirax Midi digital camera (Zeiss Mirax Midi, Zeiss, Germany).

**Figure 1:**
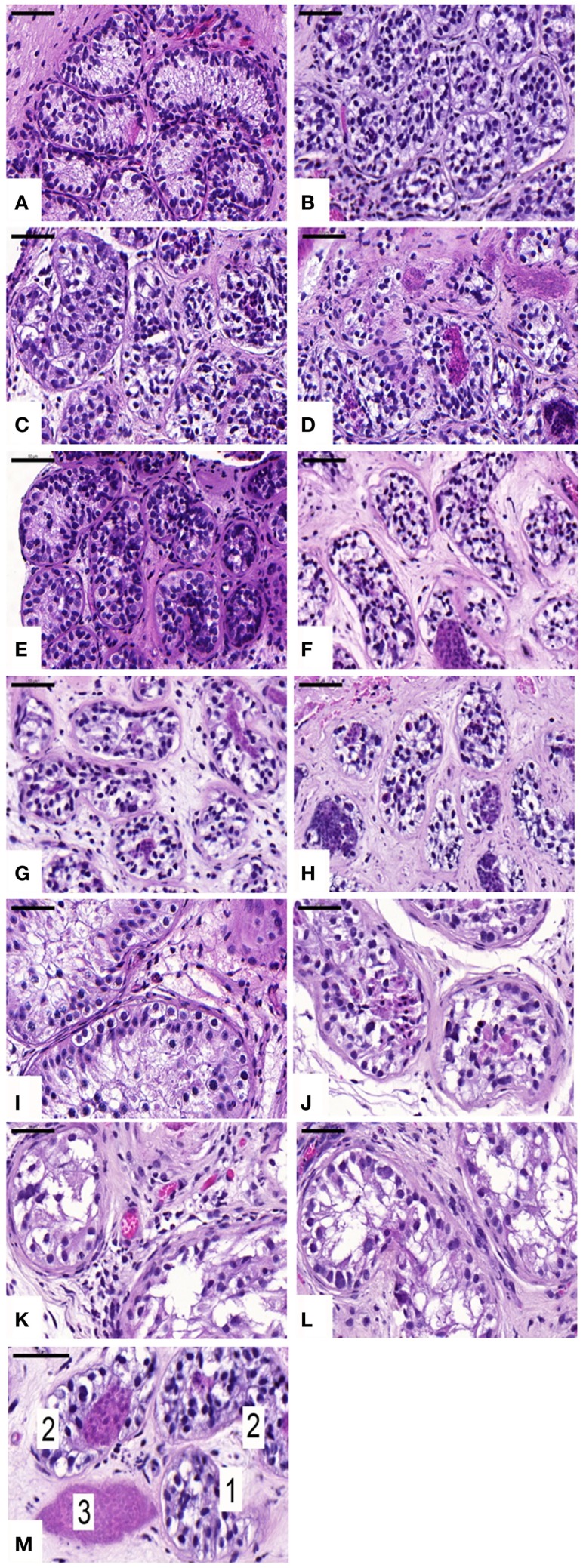
**Histological appearance and seminiferous tubule (ST) scoring**. Ungrafted controls are shown in **(A**,**E,I)**. Slow-frozen grafted tissue (group A) is shown in **(B**,**F,J)**. Slow-frozen tissue supplemented with NAC (group B) is shown in **(C**,**G,K)**. Slow-frozen tissue supplemented with testosterone (group C) is shown in **(D**,**H,L)**. Immature testicular tissue is from boys of 2 **(A–D)**, 8 **(E–H)**, and 14 years of age **(I–L)**. STs considered intact score 1, STs considered satisfactory score 2, and damaged STs score 3 [shown in **(M)**]. Scale bar: 50 μm.

Seminiferous tubule (ST) integrity was evaluated on HE-stained sections under a light microscope at 400× magnification. Tubules were considered intact (score 1) when good adhesion of cells to the basement membrane, good cell cohesion, and no sclerosis were noted. Tubules were considered satisfactory (score 2) when intratubular cells could still be individualized, despite the presence of focal necrosis. Finally, tubules were considered damaged (score 3) when complete necrosis was observed (Figure 1M).

#### Immunohistochemistry

Melanoma-associated antigen 4 (MAGE-A4) mouse anti-human monoclonal antibody was used to evidence SG. This antibody, purified from hybridoma 57B, was kindly provided by Giulio Spagnoli MD (University of Basel, Switzerland) (29).

Ki67 mouse anti-human monoclonal antibody (DAKO M7240) was used to evaluate intratubular proliferation. Ki67 is a nuclear antigen associated with cell proliferation and is present throughout the active cell cycle (late G1, S, G2, and M phases), but absent in resting cells (G0) (30).

Proliferating SG were assessed on serial sections stained for MAGE-A4 and Ki67.

Cleaved caspase-3 rabbit anti-human polyclonal antibody (Promega G7481) was used to evidence apoptotic cells resulting from both intrinsic and extrinsic apoptotic routes (31).

Cleaved caspase-8 rabbit anti-human monoclonal antibody (Cell Signaling 9496) was used to evidence the extrinsic apoptosis pathway (32).

For immunostaining, sections were deparaffinized and rehydrated. Endogenous peroxidase activity was blocked by incubating the sections with 0.3% H_2_O_2_ for 30 min at room temperature (RT).

After washing under deionized water, sections were placed in citrate buffer for 75 min at 98°C (antigen retrieval was performed for all antibodies), followed by incubation at RT with 10% normal goat serum (NGS, Invitrogen, Merelbeke, Belgium) and 1% bovine serum albumin (BSA, Invitrogen) to block non-specific binding sites for 30 min (for MAGE-A4 and Ki67).

The primary antibody (diluted to 1/500 for MAGE-A4, 1/150 for Ki67, 1/100 for caspase-8, and 1/200 for caspase-3) was added and incubated overnight at 4°C in a humidified chamber. The next day, secondary anti-mouse or anti-rabbit antibody (EnVision + System-labeled polymer-HRP; DAKO K4001-K4009) was added and incubated for 60 min at RT. Diaminobenzidine (DAKO K3468) was used as a chromogen. Nuclei were counterstained with Mayer’s hematoxylin [for detailed protocol, see Ref. (8)]. For immunohistochemistry, STs with scores 1 and 2 were evaluated.

Terminal deoxynucleotidyl transferase-mediated dUTP nick end labeling was used to detect deoxyribonucleic acid (DNA) fragmentation, found in apoptotic as well as necrotic cells. Sections were dewaxed, rehydrated, and washed in deionized water. The slides were then pretreated with 20 mg/ml of proteinase K solution (Roche Applied Science 745723) in 10 mM Tris–HCl (pH 7.5) for 30 min at 37°C in a humidified chamber. DNA strand breaks occurring during the apoptotic process were detected using the *in situ* Cell Death Detection Kit, TMR Red (Roche Applied Science 2156792). After washing with PBS, the slides were incubated with the TUNEL reaction mixture – 50 μl enzyme solution (terminal deoxynucleotidyl transferase) and 450 μl label solution (nucleotide mixture in reaction buffer) – for 60 min at 37°C in a humidified chamber protected from light, followed by rinsing in PBS. They were covered with mounting medium with 4,6-diamino-2-phenylindole (DAPI) (Vector Laboratories). This special formulation is intended to preserve fluorescence during prolonged storage and, at the same time, counterstain DNA by means of DAPI. Human tonsil tissue was used as a positive control, and negative control sections were incubated with label solution without enzyme solution. Slides were coverslipped and sealed around the perimeter with nail polish, stored at 4°C, and protected from light until examination.

TUNEL-stained and DAPI-counterstained slides were examined under an inverted fluorescence microscope (Leica; Van Hopplynus Instruments). Red fluorescence was visualized in TUNEL-positive cells using an excitation wavelength in the 520–560 nm range and by observing the emitted light at a wavelength between 570 and 620 nm. DAPI reached excitation at ±360 nm, and emitted at ±460 nm when bound to DNA, producing blue fluorescence in all nuclei.

### Statistical analysis

Analyses were performed using the JMP 7 program based on SAS. Data are presented as mean ± SD. Statistical significance between variables was evaluated using the Mann–Whitney *U*-test. A *p*-value of ≤0.05 was considered statistically significant. Comparisons were made between groups (control versus each grafting group and between grafting groups).

## Results

### Graft recovery

The graft recovery rate after 5 days of xenotransplantation was 100%.

### Histological evaluation

An average of 465 ± 299, 965 ± 433, 897 ± 777, and 774 ± 554 STs were examined on HE sections in non-grafted controls and A, B, and C grafted groups, respectively. In ungrafted controls, all STs were considered intact (score 1). In all, 21, 18, and 21% of STs showed good integrity (score 1), 67, 78, and 69% showed satisfactory integrity (score 2), and 12, 4, and 10% were damaged (score 3) in groups A, B, and C, respectively. ST integrity was not statistically different between the three grafted groups, but a significant decrease in STs with good integrity was noted between grafted tissue and ungrafted control tissue (Figure 1).

### Immunohistochemistry

#### Spermatogonial cells

**Figure 2:**
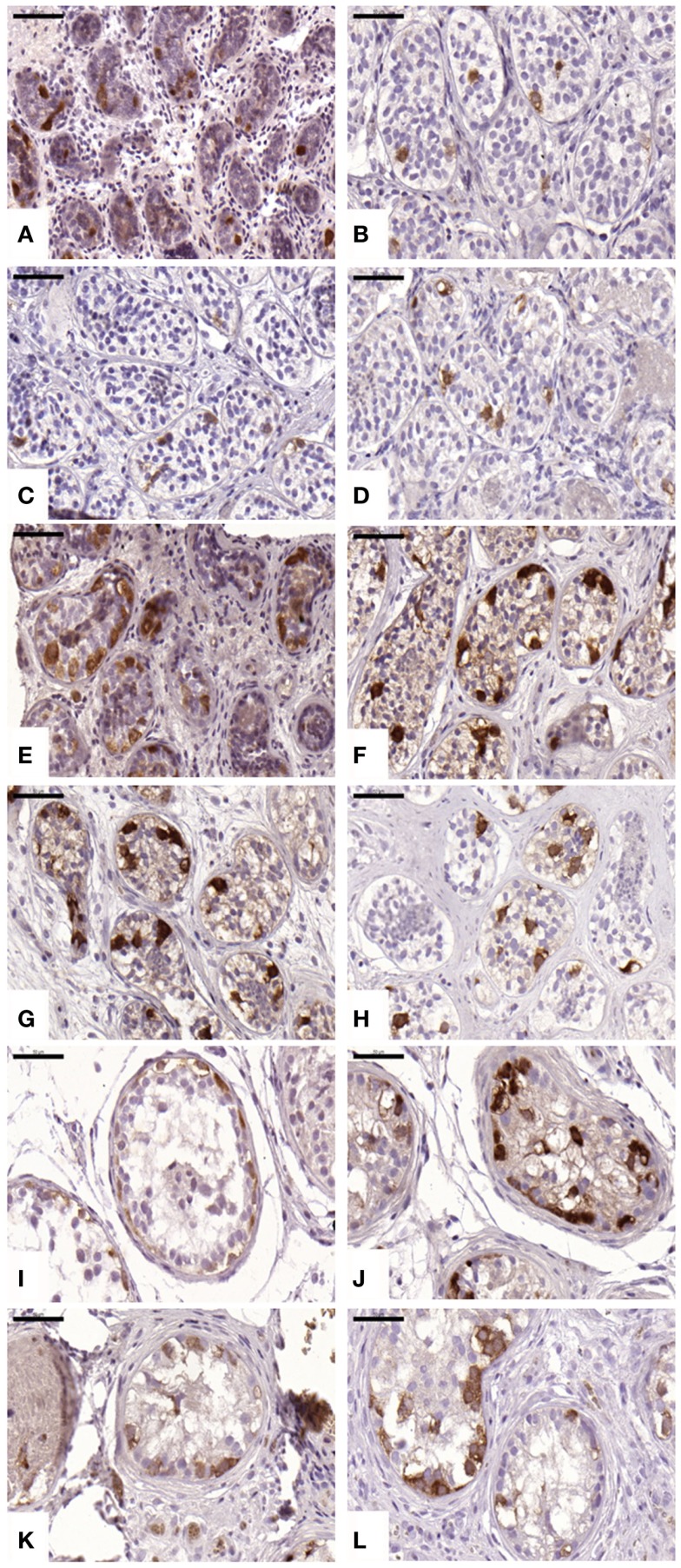
**Presence of spermatogonial cells evidenced by MAGE-A4 antibody**. Ungrafted controls are shown in **(A,E,I**). Slow-frozen grafted tissue (group A) is shown in **(B,F,J)**. Slow-frozen tissue supplemented with NAC (group B) is shown in **(C,G,K)**. Slow-frozen tissue supplemented with testosterone (group C) is shown in **(D,H,L)**. Immature testicular tissue is from boys of 2 **(A–D)**, 8 **(E–H)**, and 14 years of age **(I–L)**. Scale bar: 50 μm.

An average of 216 ± 51, 799 ± 444, 690 ± 679, and 481 ± 309 STs were analyzed in non-grafted controls and A, B, and C grafted groups, respectively. SG was identified in all grafts, as evidenced by MAGE-A4-positive cells. The mean number (Table 1) of SG per analyzed tubule (scores 1 and 2) was not significantly different between grafted groups (*p* ≥ 0.05) or between grafted groups and ungrafted controls (*p* ≥ 0.05) (Figure 2). The SG recovery rate was 67, 63, and 53% in groups A, B, and C, respectively.

**Table 1:** **Spermatogonial recovery**.

Age (years)	Ungrafted control	A	B	C
14	3.17	3.43	2.94	5.01
8	5.46	2.19	2.70	0.80
9	2.28	2.26	1.33	1.82
15	5.46	4.14	3.84	1.30
2	0.51	0.11	0.25	0.19
2	1.26	0.10	0.41	0.50
Mean ± SD	3.02 ± 2.09^a^	2.04 ± 1.67^a^	1.91 ± 1.47^a^	1.60 ± 1.77^a^

*A = slow-frozen and grafted, B = slow-frozen and supplemented with NAC, C = slow-frozen and supplemented with testosterone*.

*^a^No statistical difference was observed between groups (*p* ≥ 0.05)*.

#### Intratubular proliferative activity

**Figure 3:**
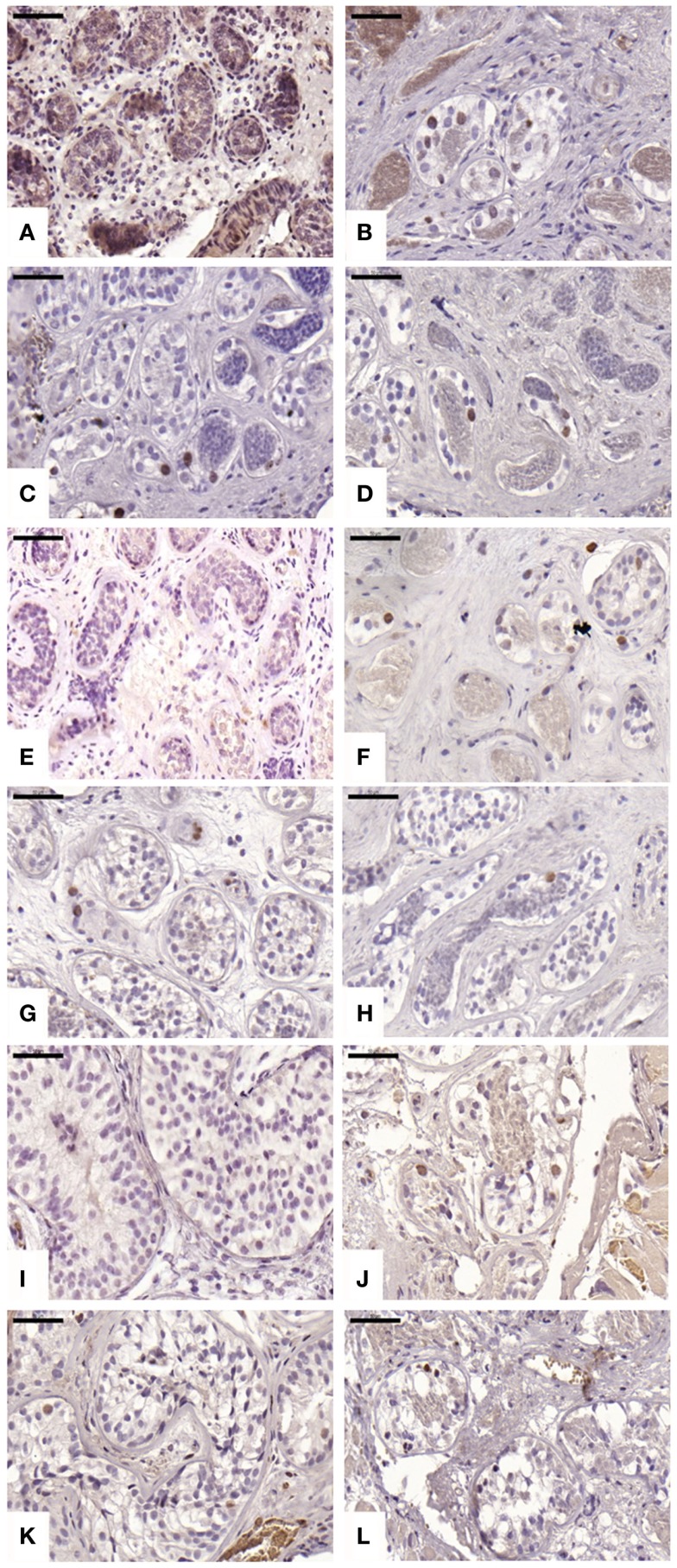
**Proliferative activity evidenced by Ki67 antibody**. Ungrafted controls are shown in **(A,E,I)**. Slow-frozen grafted tissue (group A) is shown in **(B,F,J)**. Slow-frozen tissue supplemented with NAC (group B) is shown in **(C,G,K)**. Slow-frozen tissue supplemented with testosterone (group C) is shown in **(D,H,L)**. Immature testicular tissue is from boys of 2 **(A–D)**, 8 **(E–H)**, and 14 years of age **(I–L)**. Scale bar: 50 μm.

An average of 220 ± 71, 847 ± 422, 628 ± 632, and 619 ± 481 STs were analyzed in non-grafted controls and A, B, and C grafted groups, respectively. We observed proliferation in all grafts; this proliferative activity was similar (*p* ≥ 0.05) between ungrafted controls and group B, but different in groups A and C (*p* ≤ 0.05), with a mean ± SD of proliferating cells per ST of 0 ± 0, 0.06 ± 0.12, 0.01 ± 0.01, and 0.04 ± 0.06 in non-grafted controls, slow-frozen grafted tissue, NAC grafted tissue, and testosterone grafted tissue, respectively (Figure 3). In addition, only 2.26, 0, and 3.2% of proliferating cells were SG in A, B, and C grafted groups, respectively, the remaining proliferating cells being Sertoli cells.

#### Intratubular apoptosis and TUNEL

**Figure 4:**
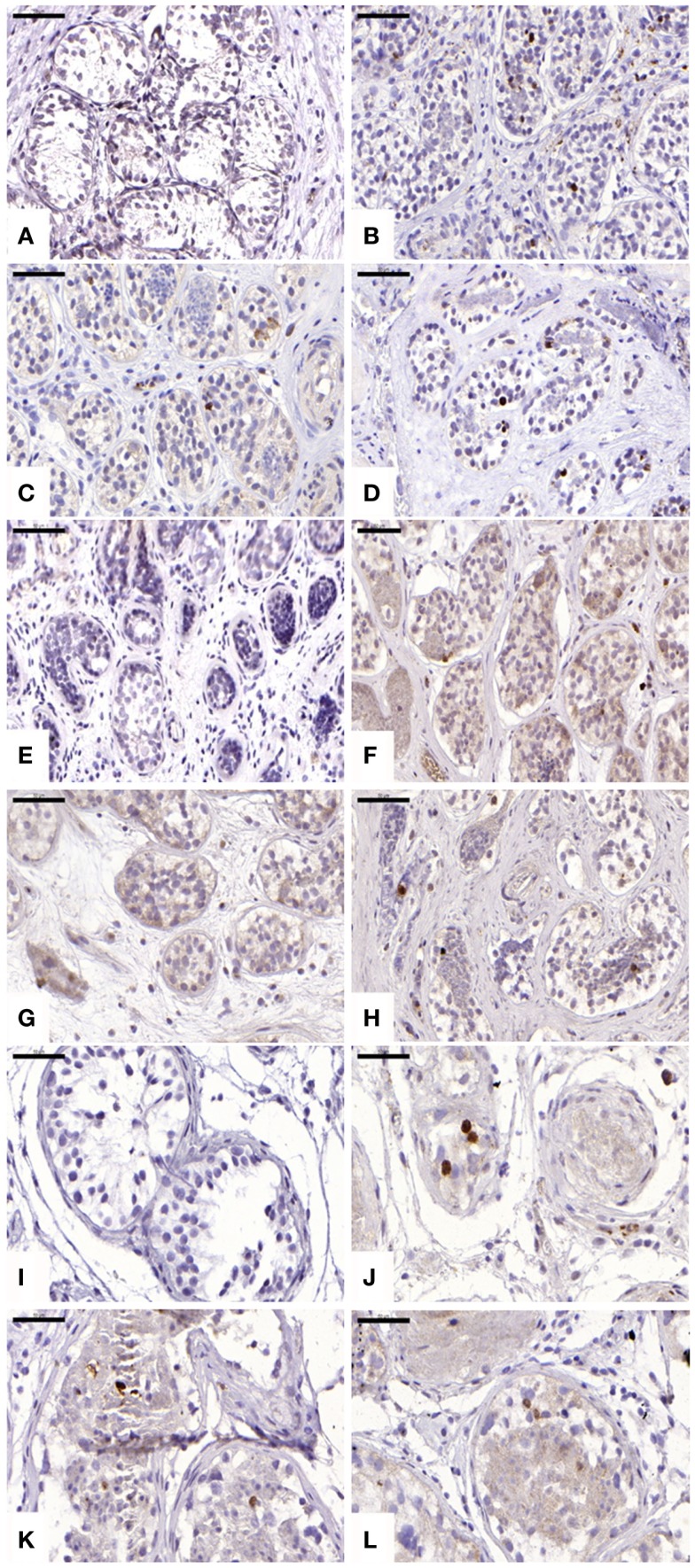
**Apoptotic activity evidenced by cleaved caspase-3 antibody**. Ungrafted controls are shown in **(A,E,I)**. Slow-frozen grafted tissue (group A) is shown in **(B,F,J)**. Slow-frozen tissue supplemented with NAC (group B) is shown in **(C,G,K)**. Slow-frozen tissue supplemented with testosterone (group C) is shown in **(D,H,L)**. Immature testicular tissue is from boys of 2 **(A–D)**, 8 **(E–H)**, and 14 years of age **(I–L)**. Scale bar: 50 μm.

For cleaved caspase-3 evaluation, an average of 107 ± 34, 184 ± 68, 146 ± 116, and 143 ± 89 STs were analyzed in non-grafted controls and A, B, and C grafted groups, respectively. Cleaved capsase-3 activity was similar (*p* ≥ 0.05) between groups, showing positive staining (0.21 ± 0.25, 0.11 ± 0.14, and 0.11 ± 0.07 cells) in groups A, B, and C, respectively (Figure 4). No staining was found in non-grafted controls. In addition, within the total number of STs analyzed (score 1 and 2), 11.65 ± 6.75%, 10 ± 9.75%, and 8.72 ± 4.77% showed cleaved caspase-3 activity, with no statistically significant difference between grafted groups (*p* ≥ 0.05).

For cleaved caspase-8 evaluation, no immunostaining was evidenced in any of the groups. Positive and negative controls were performed on human lymph nodes.

For TUNEL, an average of 104 ± 21, 74 ± 27, 54 ± 30, and 40 ± 17 STs were analyzed in non-grafted controls and A, B, and C grafted groups, respectively. A similar mean value (*p* ≥ 0.05) of 75.26 ± 20.61%, 70.60 ± 12.81%, and 83.35 ± 5.76% of positive STs were observed in A, B, and C grafted groups, respectively. A statistically significant difference between cleaved caspase-3-positive tubules and TUNEL-positive tubules was observed in all grafted groups (*p* ≤ 0.05).

## Discussion

Although still experimental, cryopreservation of ITT is a promising technique to preserve fertility in children. In animals, cryopreservation by slow-freezing or vitrification has proved effective in reinitiating spermatogenesis after tissue transplantation (33–43) and yielded healthy offspring in some species (35, 36, 41, 43). By contrast, while survival of human ITT after cryopreservation has been demonstrated in several studies (4–8, 44, 45), SG recovery was very low and no normal differentiation was achieved after several months of transplantation (5–8).

In a previous study, we compared two ITT cryopreservation protocols: slow-freezing and vitrification. Both showed equivalent results in terms of SG survival and maturation up to the pachytene stage. Unexpectedly, SG loss was similar in non-cryopreserved tissue after transplantation (8), suggesting that there may be other causes of poor transplantation outcome apart from the cryopreservation protocol applied.

While a significant decrease in the number of STs with good integrity and in SG recovery was noted between the non-grafted control group and all grafted groups, the number of SG per ST with good integrity in the grafted groups was not different from the ungrafted control group, suggesting irreversible damage to some STs. The SG recovery rate of 53–67% after 5 days is consistent with the progressive loss seen in our previous studies, where 14.5 and 3.7% of residual SG were observed after 3 weeks and 6 months of xenografting, respectively (4, 5, 8). It, therefore, appears that most SG loss occurs early during the grafting process, and hence before revascularization at 5 days and stabilization of neovessels at 21 days (11).

Interestingly, ST integrity did not worsen over time concomitantly with SG loss. Indeed, 5 days after grafting, only 18.6–21% of STs appeared to be intact, by contrast with our previous studies in the same model (4, 5), where the percentage of intact STs was not statistically different from ungrafted controls (82.19 ± 16.46% of intact STs at 3 weeks). We may, therefore, hypothesize that STs partially recover from the initial insult, but that only empty SSC niches remain or that there are some irreversible effects on SSC potential (i.e., their renewal capacity).

To support the latter hypothesis, we previously observed that intratubular proliferation was mainly due to Sertoli cell proliferation and not SG (98.87 versus 1.13% proliferating SG) 3 weeks after xenografting (4). Consistent with these data, we found that the majority of proliferating cells in grafted tissue were Sertoli cells, with 97.74, 100, and 96.8% proliferating Sertoli cells compared to 2.26, 0, and 3.2% proliferating SG for groups A, B, and C, respectively.

This could imply that either induced ischemic cellular stress and/or an inappropriate graft environment could potentially enhance recruitment of SSCs entering into the differentiation process, leading to depletion of the pool of SSCs capable of auto-renewal, which may be associated with the “burn-out” theory suggested for loss of the follicular reserve after cellular stress induced by chemotherapy in the ovary (46). An important issue to determine is to what extent this process occurs and if we may limit SG depletion by protective molecule supplementation.

We observed a slight but statistically significant increase in intratubular proliferation (Ki67) in slow-frozen controls and testosterone-supplemented grafts compared to ungrafted controls. However, for the group supplemented with NAC, this finding was not statistically significant, confirming the efficacy of our supplementation, since NAC is a known inhibitor of cell proliferation (13, 47, 48).

While it is known that maintenance and development of the graft depend on the hormonal environment (20), the influence of testosterone on xenograft outcome has not yet been investigated. *In vitro* culture of ST sections supplemented with testosterone showed suppression of apoptosis in human germ cells, confirming the crucial role of testosterone in germ cell survival (26). Furthermore, in the culture of segments of STs, testosterone withdrawal coincided with a progressive increase in the proportion of Sertoli cells showing apoptosis-related DNA fragmentation, independent of the addition of FSH (49). However, in our study, *in vivo* supplementation of xenografts with testosterone did not provoke any decrease in intratubular apoptosis.

Evaluation of apoptosis by detection of activated caspase-3 revealed a significant increase in positive cells in all three grafted groups compared to the ungrafted control group. Interestingly, a statistically significant difference between cleaved caspase-3-positive tubules and TUNEL-positive tubules was observed in each grafted group (*p* ≤ 0.05). With TUNEL staining shown to be predominant over cleaved caspase-3, we can conclude that necrosis is responsible for most of the irreversible damage to STs and SG, rather than apoptosis. ST necrosis due to initial hypoxia, as opposed to apoptosis, appears to be implicated in low SG recovery.

Addition of NAC at the tested concentration failed to demonstrate any beneficial effect on SG or ST preservation. By contrast, two studies have shown an anti-apoptotic effect of NAC in testicular tissue. Indeed, administration of NAC by IP injection in rats prevented male germ cell death from exposure to toxic methoxyacetic acid (50) and reduced apoptosis in mice when apoptosis was induced by human chorionic gonadotropin (51). The difference between these study results may be attributed to the absence of initial vascularization of the ITT, since transplantation was achieved without vascular anastomosis in our study and was thus dependent on neoangiogenesis, or a more limited anti-proliferative effect of NAC on SG cell proliferation in previous studies. We cannot fully ascertain the absence of effect at a higher concentration, as increased levels were demonstrated to have an impact in the presence of a vascular network.

We hypothesize that oxidative stress during the avascular period could also induce Sertoli cell injury and consequently lead to germ cell apoptosis. It has indeed been demonstrated that Fas expression and apoptosis in germ cells were increased after ischemia/reperfusion and that injection of anti-Fas antibody proved effective in decreasing apoptosis in these cells (52).

Moreover, Sertoli cell injury by exposure to phthalates induces germ cell apoptosis through the Fas/Fas ligand interaction (53). To further investigate apoptotic pathways implicated in SG/ST apoptosis, this extrinsic apoptotic route was analyzed by evidencing activation of caspase-8, this protein being the first activated caspase in the extrinsic pathway through the Fas/Fas ligand interaction, where Fas ligand is produced by Sertoli cells (31). Since none of our transplants showed caspase-8 activity, there is so far no evidence that Sertoli cells are directly implicated in SG loss in slow-frozen tissue grafts.

In conclusion, the results obtained in our study did not show any clear impact of the addition of NAC at the tested concentration nor testosterone on the survival of germ cells or inhibition of apoptosis in grafted ITT. The transplantation model used may play a part in this and constitutes a limitation of the study.

Although we found inhibition of SG proliferation in grafts supplemented with NAC, confirming its effect on grafted tissue, the time it takes to reach the graft or its concentration in the graft could be insufficient before reestablishment of vascularization. Therefore, since *in vitro* experiments have shown the effect of NAC on hypoxia to be dose dependent (54), increasing the doses may be considered.

Furthermore, since angiogenesis is not completed before day 21 (11), ways of obtaining better distribution of protective molecules, for more localized action at the tissular level until angiogenesis is complete, should be investigated.

Further studies to improve transplantation outcomes are, therefore, warranted before clinical autotransplantation of stored ITT can be contemplated.

## Author Contributions

Jonathan Poels performed experiments and data analysis and wrote the manuscript. Gaël Abou-Ghannam performed experiments and data analysis and wrote the manuscript. Sophie Herman assisted in performing experiments and data analysis. Anne Van Langendonckt revised the manuscript. Francois-Xavier Wese performed surgical biopsies and was responsible for critical review of the manuscript. Christine Wyns was responsible for critical review of the manuscript and the discussion.

## Conflict of Interest Statement

The authors declare that the research was conducted in the absence of any commercial or financial relationships that could be construed as a potential conflict of interest.
